# Differences in the Association between Physical Activity and People’s Resilience and Emotions during Two Consecutive Covid-19 Lockdowns in Israel

**DOI:** 10.3390/ijerph182413217

**Published:** 2021-12-15

**Authors:** Sima Zach, Sigal Eilat-Adar, Miki Ophir, Avital Dotan

**Affiliations:** The Wingate Institute, The Academic College at Wingate, Netanya 4290200, Israel; ophir-m@wincol.ac.il (M.O.); avital@tandu.co.il (A.D.)

**Keywords:** COVID-19, physical activity, resilience, emotions, depression, lockdown

## Abstract

Governments worldwide have imposed harsh restrictions for decreasing the Covid-19 pandemic and maintaining public health. Yet such limitations have impacted people’s physical activity. This study examined relationships between changes in physical activity and resilience, emotions, and depression during two lockdowns in Israel. An online survey was completed twice by 135 participants during two consecutive lockdowns. The results indicate that resilience and positive emotions were higher, and negative emotions and depression were lower during the second lockdown compared to the first one—even though people spent less time performing physical activity in the later lockdown. Moreover, negative emotions significantly decreased among people who reported increased physical activity during the second lockdown [M = 2.2 (SD = 0.9) compared to M = 1.9 (SD = 0.8) on a scale of 1–5] and increased among those who reported a reduction in activity [M = 1.8 (SD = 0.7) compared to M = 2.2 (SD = 0.7)]. It could therefore be concluded that while the Israeli population’s resilience is higher compared to other populations (who do not regularly deal with crisis situations), their increased physical activity was associated with better resilience and emotions and lower depression scores. Since lockdowns are an extreme yet often repeated phenomenon, it is important to understand the psychological implications of engaging in physical activity.

## 1. Introduction

In March 2020, COVID-19 was declared a global pandemic by the World Health Organization (WHO) (https://www.who.int/docs/default-source/coronaviruse/transcripts/who-audio-emergencies-coronavirus-press-conference-full-and-final-11mar2020.pdf, accessed on 9 December 2021). With governments around the globe placing unprecedented restrictions on people’s daily lives, people’s physical habits were also affected—during the pandemic in general and lockdown in particular [1]. For example, in a survey among 255 UK students, their physical activity (PA) had decreased from 223 to 173 min per week since the pandemic outbreak, with an even more pronounced reduction during lockdowns, when PA participation was hindered due to restrictions [2].

Several reasons have been noted for decreased PA during the pandemic, including personal barriers, lack of motivation, and lack of appropriate facilities, equipment, and space [3]. In a systematic review of 64 studies with a total of 86,981 participants [4], PA changes were reported, most presenting an increase in sedentary behaviors and a decrease in PA levels. Moreover, in a survey completed by 35 research organizations in Europe, North Africa, the Americas, and Western Asia, home confinement due to Covid-19 was found to be associated with reduced PA intensity (i.e., overall PA, walking, moderate, and vigorous PA), and with an increase in sitting time from five to eight hours per day [5]. However, the American College of Sports Medicine (ACSM) recommends that adults engage in moderate-intensity cardiorespiratory exercises for at least 150 min per week, and that vigorous-intensity cardiorespiratory exercise training should be performed at least 75 min per week [6].

The beneficial effects of PA on both physical outcomes [7] and psychological ones [8] have been demonstrated extensively. For example, a meta-analysis of randomized controlled trials and prospective observational studies [9] demonstrated that higher levels of habitual PA are associated with a 31% risk reduction in community-acquired infectious diseases (*n* = 557,487), and with a 37% risk reduction in infectious disease mortality (*n* = 422,813). Antibody concentration after vaccination was also higher when an adjunct PA program was in place 6 studies (*n* = 497).

Correlations between PA and psychological distress has also examined during the COVID-19 pandemic. In a study of 1332 adults in Portugal (aged 18–55), those who reported participating in more PA than usual during lockdown presented lower levels of anxiety. Moreover, participants who performed moderate and high PA exhibited lower levels of anxiety and related characteristics compared to participants with low PA [10]. Similar results were observed in a survey with 2140 participants in Brazil [11]. A drastic reduction in mental well-being has been reported following the Covid-19 Pandemic, as seen in a survey of 14,975 individuals from 14 countries. High PA levels pre-restrictions and decreased vigorous PA during restrictions were found to be associated with decreased mental and physical well-being [12]. On the other hand, increased moderate PA (~10min/day) during leisure time, from week two to week four of lockdown, was associated with improved physical and mental health [13]. Similar results were reported by [14].

Several studies that compared health behaviors between lockdowns found that such behaviors had worsen during the second lockdown compared to the first one, and that people were more concerned about their family members contracting the Coronavirus in the later Covid-19 wave. For example, in Austria, higher consumption levels of alcoholic beverages were reported during the second lockdown [15], and higher levels of anxiety and depressive symptoms were reported in the second wave by French university students [16]. In a large-scale study (*n* = 2013) conducted in Italy, poor sleep and severe depression during the second wave were reported [17], and in another Italian survey, increased daytime dysfunctions due to sleep disruptions and later sleeping times were seen during the second lockdown compared to the first one [18].

As such, it could be summarized that COVID-19 is associated with higher distress and lower well-being, which may not be surprising as the pandemic has reduced people’s PA, which is associated with worsening mental health. However, despite concerns regarding the effects of several repeated lockdowns [19] as examined in the studies described above, to the best of our knowledge, associations between changes in PA during repeated lockdowns and mental well-being have not yet been investigated in Israel. Moreover, as evidence-based data may not always reflect that of other countries [20], this study examined the relationships between the PA behaviors and mental wellbeing in Israel during two consecutive lockdowns.

More specifically, these two consecutive lockdowns were examined from several angles: (1) describing participants’ PA habits during the two lockdowns; (2) comparing their PA levels with published recommendations; (3) examining changes to PA behavior in the two lockdowns (4) assessing changes in resilience, emotions, and depression between the two lockdowns; and (5) comparing resilience, emotions, and depression according to the presented PA changes.

## 2. Methods

### 2.1. Study Design and Participants

In this study, the analysis is based on a nonprobability snowball sampling strategy, in line with reports of a higher response rate reported using online snowball surveys compared to other strategies [21]. The study was conducted during the first and second lockdowns enforced by the Israeli government: 14 March–April 2020 (first wave) (https://www.gov.il/en/departments/news/11032020_21, https://www.gov.il/en/departments/news/15032020_01_01, accessed on 9 December 2021); 25 September–17 October (second wave) (https://www.gov.il/en/departments/news/24092020_01, accessed on 9 December 2021). The questionnaire was distributed via social media, and to prevent a biased sample, respondents were asked to share the questionnaire with their contacts and ask them to do the same.

Our inclusion criteria referred to participants aged 18 and above, from anywhere in Israel. In order to examine the research objectives, although 1855 participants complete our survey during the first wave, only those who also did so during the second survey were included in the final study. G-Power analysis 3.14 [22] for analysis of variance (ANOVA) with repeated measures (RP), within-between interaction and a small effect size *f*^2^(*v*) = 0.04, *α* = 0.05, and power (1 − *β*) = 0.85, resulted in a sample size of *n* = 72, with a critical *F* = 3.12. In total, 135 Israeli participants from were eligible for inclusion, after completing the survey in both the first and second lockdowns. The sample included 42 men (31%) and 93 women (69%). The participants had an average age of 48.9 years (SD = 14.5), average body mass index (BMI) of 25.1 Kg/m^2^ (SD = 4.3).

### 2.2. The Assessment

The questionnaire consisted of six sections that were translated from English into Hebrew and then back-translated by three professional translators, in line with recommendations of other cross-cultural researchers [23]: (1) Demographic questionnaire; (2) The International Physical Activity Questionnaire (IPAQ); (3) Positive and Negative Affect Schedule (PANAS); (4) The Connor–Davidson Resilience Scale (CD-RISC); (5) Questionnaire for measuring depressive moods; and (6) Weight change, based on BMI.

A demographic questionnaire was used to receive background data regarding the participants.

A short version of the IPAQ [24] was implemented to assess the participants’ PA during the previous week, including the frequency, duration, and intensity of their different PAs.

A confirmatory factor analysis was conducted to reconfirm the behavior factors for the entire current cohort. The Kaiser-Guttman rule (eigenvalue > 1) and the scree plot were used to define the number of factors. Two factors were created for the resilience scale, one for the depression scale, and two for the PANAS scale with eigenvalues values > 1 [14].

Using the PANAS, participants were asked to rate their emotions during the past month on a scale of 1 (hardly or not at all) to 5 (to a great extent). This section included 10 questions about positive feelings (e.g., feeling strong or inspired), and 10 questions regarding negative feelings (e.g., feeling worried or guilty). Cronbach alphas for the original scale were 0.89 for a positive effect and 0.92 for a negative effect, showing high reliability [25]. The Hebrew version of this scale ranged from alphas 0.80 to 0.91 [26].

Using the CD-RISC by [27], participants were asked to rate 25 items on a scale of 0 (no resilience at all) to 4 (great resilience). The original validation of internal consistency was 0.89; Reliability assessed through the test-retest method exhibited high levels of agreement with an intraclass correlation coefficient of 0.87. Two factors created by factor analysis were used in the current study: (1) Positive acceptance of change, and (2) Personal ability, self-competence, and self-control

In this section of the questionnaire, the participants were asked to rate six questions relating to depressive moods [28] on a 4-point scale. Internal consistency for this section was 0.86.

Finally, the participants were asked to provide data relating to their BMI.

All participants signed an informed consent form and did not receive any monetary compensation for their participation. The study was approved by the Ethics Committee at the authors’ academic college, Approval #250.

After calculating the participants’ daily PA in minutes multiplied by the number of days each week, the results were divided into three categories: light, medium, and intense weekly PA. The medium and intense categories were summed together, with each minute of intense PA being calculated as two minutes of moderate activity.

### 2.3. Statistical Analysis

The participants were divided into three groups based on the amount of their moderate to vigorous (MVPA) PA during the given periods: (a) completely inactive; (b) less than the recommended quantity of PA; and (c) as recommended (or more) by the American College of Sports Medicine [6]. *χ*^2^ test was used to compare between the waves.

Next, we divided the participants into three groups according to their PA behavior patterns: (a) decreased PA; (b) no change; and (c) increased PA—during the second wave compared to the first one.

Each dependent variable was examined for normality assumption via skewness (*SK* < [2.0]) and kurtosis (*K* < 7.00) procedures. Skewness values ranged between −0.69 to 1.01 Kurtosis values ranged between −0.42 to 1.00., therefore a normal distribution was assumed for dependent variables. ANOVA with repeated measures (Bonferroni correction) was then used to compare the emotions between the three groups. A mixed RM ANOVA (two test dates X three activity groups) analysis was used to test changes in the emotion-related variables. Tukey’s post-hoc multiple comparison tests were performed when the F-test was significant (*p* < 0.05). Significant differences are presented as Cohen’s standardized values. Cohen’s *d* < 0.30 is considered a low effect, 0.30–0.70 a moderate effect, and >0.70 a strong effect.

Data were analyzed using the SPSS version 26, by IBM Corp., Redmont, VA, USA.

## 3. Results

The descriptive data for demographic, resilience, emotions, and depression scores are summarized in Table 1. Participants were middle age on average, they had a normal average BMI and most of them were women. All scores for positive resilience and emotions were higher in the second wave that in the first one. Moreover, the mean depression factor was significantly lower during the second wave. The mean negative emotions factor, however, did not differ between waves.

The participants were divided into three categories according to the WHO’s recommendation (2020) (https://www.who.int/news-room/fact-sheets/detail/physical-activity, accessed on 9 December 2021) for performing 150 min of aerobic PA per week: did not do any PA (or less than 15 min per week), did less than the recommendation, and did at least 150 min aerobic PA per week. PA according to WHO recommendations in the first and second waves are presented in Table 2.

**Figure 1:**
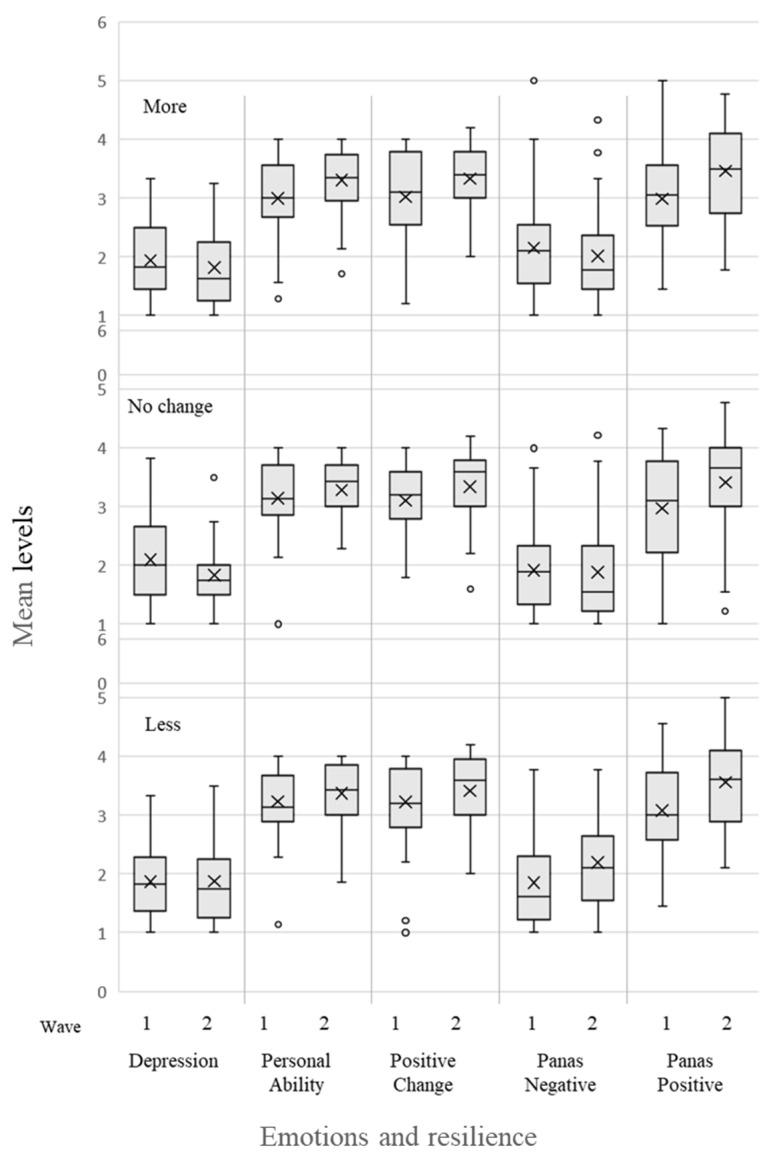
Box plot of emotion-related variables for the three PA groups in Waves 1 and 2. Mid bar = median; X = mean.

Our findings show that in both lockdowns, the majority of participants performed PA as recommended by the both the WHO and ACSM [6], with no significant difference between the two waves. PA differences between the second and the first waves were distributed normally, with the average difference in the number of minutes being M = −74.8 (SD = 5.3). In other words, decreased PA time was seen during in the second wave. Average differences were calculated for the three ACSM recommendation categories. Almost equal distribution was observed in three categories of PA difference between waves: 43 (31.9%) of participants increased their PA in the second wave, 49 (36.3%) didn’t change their PA in the second wave, and 43 (36.3%) did less PA in the second wave. Resilience, emotions, and depression scores in waves 1 and 2, according to the three PA difference categories and the interaction between group and time, are summarized in Table 3 and presented as a box plot in Figure 1. Significant differences are presented as Cohen’s standardized values. Cohen’s d < 0.30 is considered low effect, 0.30–0.70 moderate effect, and >0.70 a strong effect.

The mean resilience factors of positive acceptance of change and personal ability were higher in all categories of PA during the second wave, with no differences between groups and no interaction between time and category. For Positive acceptance of change, *F*_(2,121)_ = 0.30, *p* = 0.74, *ƞ*^2^ = < 0.01, and for Personal ability, *F*_(2,121)_ = 1.21, *p* = 0.33, *ƞ*^2^ = 0.02.

**Figure 2:**
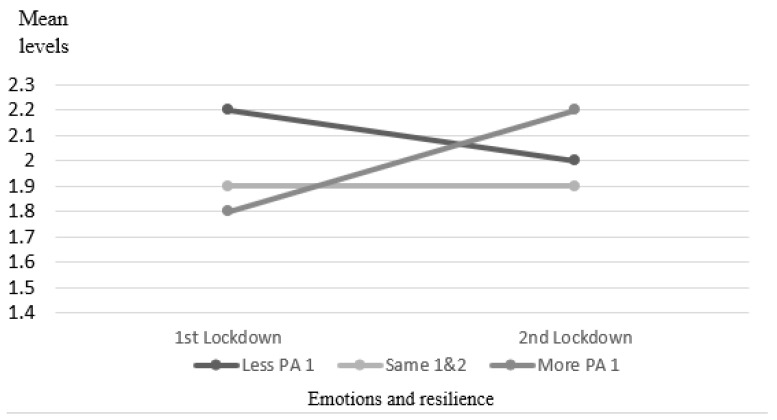
Mean negative emotions in waves 1 and 2.

Moreover, positive emotions were higher in all three PA categories during the second wave, with no significant difference between groups and no interaction between time and category, *F*_(2,118)_ = 0.023, *p* = 0.98, *ƞ*^2^ < 0.01. Negative emotions were lower among those who increased PA during the second wave compared to the first wave, and significantly higher among those who decreased their PA *F*_(2,118)_ = 4.856, *p* = 0.009, *ƞ*^2^ = 0.03. Therefore, a significant interaction was observed between groups and time, as presented in Figure 2.

**Figure 3:**
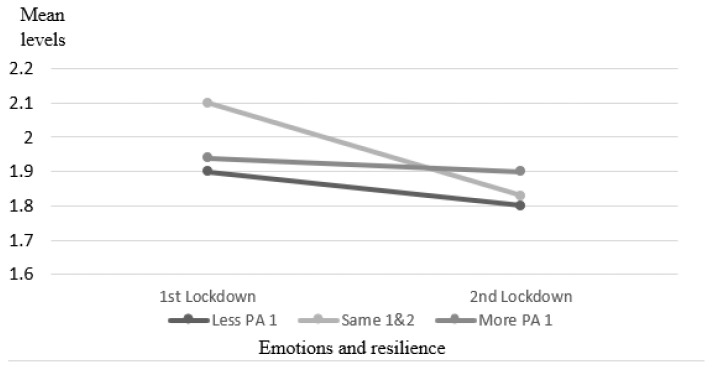
Mean depression levels in waves 1 and 2.

The depression score was different between the two waves; it was significantly lower in the second wave only among those who had no change in PA. However, this group had the highest depression level in wave 1. There was a significant difference between times with no change and lower depression scores in all groups in the second wave. There was no interaction, and also no significant difference between the three groups, *F*_(2,121)_ = 1.73, *p* = 0.18, *ƞ*^2^ = 0.03. Changes are presented in Figure 3.

## 4. Discussion

The purpose of this study was to examine PA engagement during the first and second COVID-19 lockdowns in Israel and assess the relationships between changes in PA behavior and resilience and emotional factors—based on the input provided by participants who completed the questionnaire twice.

The results show that resilience and positive feelings were higher in the second wave compared to the first one, while depression scores were lower, regardless of the participants’ PA level. These results differ from findings from other countries such as Spain and Germany. In a Spanish study, researchers assessed the change in mental health four times over a continuous strict fifty-day period of confinement and found that depressive symptoms increased between the four assessed time points (Odds ratio = 2.93, 95% confidence interval = 1.97, 4.38). Moreover, increased MVPA levels were also associated with decreased depressive symptoms [29]. While their study used the same questionnaire as in the current study [29] assessed changes in PA during one single home confinement, whereas the current study compared two consecutive yet discrete lockdowns. In a study from Germany, the psychological burden and levels of depression increased in the second phase of restrictions [30].

There may be several explanations for such differences. First, Israel placed different restrictions on the first two lockdowns in the country: While the first lockdown in Israel was very limiting in terms of outdoor activity (similar to countries such as France, Italy, New Zealand, India, and China), the second lockdown was less strict (similar to Belgium) Our ongoing list of how countries are reopening, and which ones remain under lockdown. https://www.businessinsider.nl/countries-on-lockdown-coronavirus-italy-2020-3?international=true&r=US, accessed on 9 December 2021). Second, it is also possible that the results reflect different psychologically habituation in different countries. As such, these differences may emphasize the unique nature of Israel—a country that has continuously experienced emergency situations over the years, due to social, political, and historical circumstances [31]. As such, it is possible that the population’s resilience is higher in Israel compared to other populations that have not had to regularly cope with crises. For example, during the Gulf War in 1991, Israel as a society was reported to have coped well with the stress [32]. Later, [33] reported that the psychological impact of terrorist threats over a 19-month period in Israel was moderate.

We did not find any studies on COVID-19 from additional countries that are frequently subjected to emergency situations. This hypothesis should therefore be examined in additional countries that are in a constant state of instability (such as wars, floods, and earthquakes). Another possibility is that Israel has the necessary resources for coping with such crises, as over the years, the government has built a robust, relatively efficient public health care system, with an extensive array of high-quality medical services and technologies that are readily available and are often even free of charge, based on the National Health Insurance legislation that was introduced in 1994 [34]. Moreover, the deployment of the COVID-19 vaccine in Israel was rapid and successful, due to the well-organized national efforts of all health sectors in the country, the existing infrastructure, and the allocation of resources [35].

More than half the participants engaged in PA in both waves, (as per the WHO recommendations)—a high rate that could be explained by the study sampling that was conducted by the researchers who are lecturers at a sports college. Moreover, no differences were seen in the participants’ PA levels between the two waves. The same levels of PA engagement have been reported by other researchers, such as [36], who found that of a sample of 317 Italian adults, 60.9%were highly active during lockdown. In a study by [37] that included 217 participants, being sufficiently active was reported to be higher prior to the pandemic (68.2% of the participants) compared to 60.6% during COVID-19. Finally, the 135 participants who completed our questionnaire in both waves may have been more physically active than average, rendering the sample as unrepresentative of the Israeli population.

The associations between PA changes and resilience, emotions, and depression according to PA changes are in line with the theoretical background for some but not all of the examined parameters. Negative emotions were lower among those who increased PA during the second wave compared to the first wave, and significantly higher among those who decreased their PA. Depression scores were highest in the first wave for those who presented no change in PA, but were significantly lowered in the second wave, resulting in lower depression score for all PA groups during the second wave, and with no difference between them.

The current study had several limitations. First, a snowball sampling method, conducted by researchers from a sports college, had low external validity and generalizability is not possible. Second, our study examined PA and emotions during the two initial lockdowns, which occurred within a relatively short period of time of each other. A longer timeframe may have presented greater changes in PA and emotions. Finally, our hypothesis that the results in Israel will be different from those seen in other countries needs further investigation. However, this article is unique as it compares between two lockdowns, rather than between lockdown and non-lockdown circumstances. Moreover, our findings indicate clear trends with practical implications, the study seems to have overcome these possible limitations.

## 5. Conclusions

Negative emotions and depression factors were lower during the second wave compared to the first. Moreover, in the second wave, all participants expressed higher positive emotions and resilience factors, and lower negative emotions and depression levels. Regardless, the benefits of PA during an ongoing crisis are clear. As such, stakeholders and decision makers—in the public health domain and in the education system—should enable and encourage the population to engage in regular PA, both at home and in the public domain.

## Figures and Tables

**Table 1:** Demographic and resilience, emotions, and depression during waves 1 and 2.

Variable	Factors	1st Wave	2nd Wave	*p*
		*Mean (SD) or N(%)*		
Age (years)		48.9 (14.5)		
BMI (Kg/m^2^)		25.1 (4.3)		
Men (%)		42 (31)		
Resilience	Positive acceptance of change	0.31 (0.7)	3.4 (0.6)	<0.01
	Personal ability, self-competence, and self-control	3.1 (0.5)	3.3 (0.7)	<0.01
Emotions (PANAS)	Positive	3.0 (0.8)	3.5 (0.8)	<0.01
	Negative	1.9 (0.8)	2.1 (0.8)	0.42
Depression		2.0 (0.6)	1.8 (0.6)	0.02

**Table 2:** PA * Quantities Compared to WHO Recommendations. * PA—Physical activity.

Significance	2nd Wave [*n* (%)]	1st Wave [*n* (%)]	PA Quantity
Not at all	(17.8) 24	(23.0) 31	$\chi_{\left(2\right)}^{2}=2.57$ *p* = 0.28
Less than recommended	(22.2) 30	(26.7) 36	
In line with or higher than recommendations	(60.0) 81	(50.4) 68	

**Table 3:** Resilience, Emotions, and Depression according to PA during 2nd Lockdown.

		Category I Increased PA *Mean (SD)*				Category II No Change in PA *Mean (SD)*				Category III Decreased PA *Mean (SD)*				*p* Time X Group	*p* between Times	*p* between Groups
Variable		1st	2nd	*p*	*d*	1st	2nd	*p*	*d*	1st	2nd	*p*	*d*			
Resilience	Positive acceptance of change	3.0 (0.7)	3.3 (0.6)	<0.01	0.49	3.1 (0.7)	3.3 (0.5)	0.01	0.38	3.2 (0.7)	3.4 (0.6)	0.08	0.25	00.74	<0.01	0.43
	Personal ability, self-competence, and self-control	0.30 (0.7)	0.33 (0.5)	<0.01	0.54	3.1 (0.6)	3.3 (0.5)	0.09	0.29	3.2 (0.6)	3.4 (0.5)	0.14	0.21	0.33	<0.01	0.39
Emotions (PANAS)	Positive	3.0 (0.7)	3.3 (0.6)	0.06	0.62	3.1 (0.7)	3.3 (0.6)	0.01	0.51	3.2 (0.7)	3.4 (0.6)	0.08	0.52	0.67	<0.01	0.98
	Negative	0.22 (0.9)	1.9 (0.8)	0.22	0.17	1.9 (0.7)	1.9 (0.8)	0.76	0.05	1.8 (0.7)	2.2 (0.7)	<0.01	0.52	<0.01	0.42	0.47
Depression		1.9 (0.6)	0.18 (0.6)	0.22	0.21	2.1 (0.7)	1.8 (0.6)	0.001	0.39	1.9 (0.6)	1.9 (0.7)	0.97	0.01	0.68	0.03	0.18

## Data Availability

Data is available upon request from the corresponding author.

## References

[1] Harrison E., Monroe-Lord L., Carson A.D., Jean-Baptiste A.M., Phoenix J., Jackson P., Harris B.M., Asongwed E., Richardson M.L. (2021). COVID-19 pandemic-related changes in wellness behavior among older Americans. BMC Public Health.

[2] Maltagliati S., Rebar A., Fessler L., Forestier C., Sarrazin P., Chalabaev A., Sander D., Sivaramakrishnan H., Orsholits D., Boisgontier M.P. (2021). Evolution of physical activity habits after a context change: The case of COVID-19 lockdown. Br. J. Health Psychol..

[3] Farah B.Q., Prado W.L.D., Malik N., Lofrano-Prado M.C., de Melo P.H., Botero J.P., Cucato G.G., Correia M.D.A., Ritti-Dias R.M. (2021). Barriers to physical activity during the COVID-19 pandemic in adults: A cross-sectional study. Sport Sci. Health.

[4] Stockwell S., Trott M., Tully M., Shin J., Barnett Y., Butler L., McDermott D., Schuch F., Smith L. (2021). Changes in physical activity and sedentary behaviours from before to during the COVID-19 pandemic lockdown: A systematic review. BMJ Open Sport Exerc. Med..

[5] Ammar A., Brach M., Trabelsi K., Chtourou H., Boukhris O., Masmoudi L., Bouaziz B., Bentlage E., How D., Ahmed M. (2020). Effects of COVID-19 Home Confinement on Eating Behaviour and Physical Activity: Results of the ECLB-COVID19 International Online Survey. Nutrients.

[6] Garber C.E., Blissmer B., Deschenes M.R., Franklin B.A., LaMonte M.J., Lee I.-M., Nieman D.C., Swain D.P., American College of Sports Medicine (2011). American College of Sports Medicine position stand. Quantity and Quality of Exercise for Developing and Maintaining Cardiorespiratory, Musculoskeletal, and Neuromotor Fitness in Apparently Healthy Adults: Guidance for Prescribing Exercise. Med. Sci. Sports Exerc..

[7] Lee I.-M., Shiroma E.J., Lobelo F., Puska P., Blair S.N., Katzmarzyk P.T. (2012). Lancet Physical Activity Series Working Group. Effect of physical inactivity on major non-communicable diseases worldwide: An analysis of burden of disease and life expectancy. Lancet.

[8] Rebar A.L., Stanton R., Geard D., Short C., Duncan M.J., Vandelanotte C. (2015). A meta-meta-analysis of the effect of physical activity on depression and anxiety in non-clinical adult populations. Health Psychol. Rev..

[9] Chastin S.F.M., Abaraogu U., Bourgois J.G., Dall P.M., Darnborough J., Duncan E., Dumortier J., Pavón D.J., McParland J., Roberts N.J. (2021). Effects of Regular Physical Activity on the Immune System, Vaccination and Risk of Community-Acquired Infectious Disease in the General Population: Systematic Review and Meta-Analysis. Sports Med..

[10] Frontini R., Rebelo-Gonçalves R., Amaro N., Salvador R., Matos R., Morouço P., Antunes R. (2021). The Relationship between Anxiety Levels, Sleep, and Physical Activity During COVID-19 Lockdown: An Exploratory Study. Front. Psychol..

[11] Puccinelli P.J., da Costa T.S., Seffrin A., de Lira C.A.B., Vancini R.L., Nikolaidis P.T., Knechtle B., Rosemann T., Hill L., Andrade M.S. (2021). Correction to: Reduced level of physical activity during COVID-19 pandemic is associated with depression and anxiety levels: An internet-based survey. BMC Public Health.

[12] Wilke J., Hollander K., Mohr L., Edouard P., Fossati C., González-Gross M., Ramírez C.S., Laiño F., Tan B., Pillay J.D. (2021). Drastic Reductions in Mental Well-Being Observed Globally During the COVID-19 Pandemic: Results From the ASAP Survey. Front. Med..

[13] Cheval B., Sivaramakrishnan H., Maltagliati S., Fessler L., Forestier C., Sarrazin P., Orsholits D., Chalabaev A., Sander D., Ntoumanis N. (2021). Relationships between changes in self-reported physical activity, sedentary behaviour and health during the coronavirus (COVID-19) pandemic in France and Switzerland. J. Sports Sci..

[14] Zach S., Zeev A., Ophir M., Eilat-Adar S. (2021). Physical activity, resilience, emotions, moods, and weight control of older adults during the COVID-19 global crisis. Eur. Rev. Aging Phys. Act..

[15] Łaszewska A., Helter T., Simon J. (2021). Perceptions of Covid-19 lockdowns and related public health measures in Austria: A longitudinal online survey. BMC Public Health.

[16] Charbonnier E., Le Vigouroux S., Goncalves A. (2021). Psychological Vulnerability of French University Students during the COVID-19 Pandemic: A Four-Wave Longitudinal Survey. Int. J. Environ. Res. Public Health.

[17] Salfi F., D’Atri A., Tempesta D., Ferrara M. (2021). Sleeping under the waves: A longitudinal study across the contagion peaks of the COVID-19 pandemic in Italy. J. Sleep Res..

[18] Conte F., Cellini N., De Rosa O., Rescott M.L., Malloggi S., Giganti F., Ficca G. (2021). Dissociated profiles of sleep timing and sleep quality changes across the first and second wave of the COVID-19 pandemic. J. Psychiatr. Res..

[19] Adam D. (2020). Special report: The simulations driving the world’s response to COVID-19. Nat. Cell Biol..

[20] Rajkumar R.P. (2020). COVID-19 and mental health: A review of the existing literature. Asian J. Psychiatry.

[21] Kosinski M., Matz S.C., Gosling S.D., Popov V., Stillwell D. (2015). Facebook as a research tool for the social sciences: Opportunities, challenges, ethical considerations, and practical guidelines. Am. Psychol..

[22] Faul F., Erdfelder E., Lang A.-G., Buchner A. (2007). G*Power 3: A flexible statistical power analysis program for the social, behavioral, and biomedical sciences. Behav. Res. Methods.

[23] Brislin R.W. (1970). Back-translation for cross-cultural research. J. Cross Cult. Psychol..

[24] Georgopoulos V.C., Perdikogianni M., Mouskenteri M., Psychogiou L., Oikonomou M., Malandraki G.A. (2018). Cross-Cultural Adaptation and Validation of the SWAL-QoL Questionnaire in Greek. Dysphagia.

[25] Maher J.P., Hevel D.J., Reifsteck E.J., Drollette E.S. (2021). Physical activity is positively associated with college students’ positive affect regardless of stressful life events during the COVID-19 pandemic. Psychol. Sport Exerc..

[26] Hamama L., Ronen T., Shachar K., Rosenbaum M. (2013). Links between Stress, Positive and Negative Affect, and Life Satisfaction Among Teachers in Special Education Schools. J. Happiness Stud..

[27] Connor K.M., Davidson J.R.T. (2003). Development of a new resilience scale: The Connor-Davidson Resilience Scale (CD-RISC). Depress. Anxiety.

[28] Kandel D.B. (1982). Epidemiology of Depressive Mood in Adolescents. Arch. Gen. Psychiatry.

[29] Cecchini J.A., Carriedo A., Fernández-Río J., Méndez-Giménez A., González C., Sánchez-Martínez B., Rodríguez-González P. (2021). A longitudinal study on depressive symptoms and physical activity during the Spanish lockdown. Int. J. Clin. Health Psychol..

[30] Moradian S., Bäuerle A., Schweda A., Musche V., Kohler H., Fink M., Weismüller B., Benecke A.-V., Dörrie N., Skoda E.-M. (2021). Differences and similarities between the impact of the first and the second COVID-19-lockdown on mental health and safety behaviour in Germany. J. Public Health.

[31] Davidovitz M., Cohen N. (2020). Playing defence: The impact of trust on the coping mechanisms of street-level bureaucrats. Public Manag. Rev..

[32] Milgram N. (1993). (Noach) Stress and Coping in Israel during the Persian Gulf War. J. Soc. Issues.

[33] Bleich A. (2003). Exposure to Terrorism, Stress-Related Mental Health Symptoms, and Coping Behaviors among a Nationally Representative Sample in Israel. JAMA.

[34] Clarfield A.M., Manor O., Bin Nun G., Shvarts S., Azzam Z.S., Afek A., Basis F., Israeli A. (2017). Health and health care in Israel: An introduction. Lancet.

[35] Muhsen K., Cohen D. (2021). COVID-19 vaccination in Israel. Clin. Microbiol. Infect..

[36] Guidetti M., Averna A., Castellini G., Dini M., Marino D., Bocci T., Ferrucci R., Priori A. (2021). Physical Activity during COVID-19 Lockdown: Data from an Italian Survey. Healthcare.

[37] Gierc M., Riazi N.A., Fagan M.J., Di Sebastiano K.M., Kandola M., Priebe C.S., Weatherson K.A., Wunderlich K.B., Faulkner G. (2021). Strange Days: Adult Physical Activity and Mental Health in the First Two Months of the COVID-19 Pandemic. Front. Public Health.

